# PRKACB Attenuates Chondrocyte Loss and Inflammation in Osteoarthritis

**DOI:** 10.1002/iid3.70342

**Published:** 2026-02-12

**Authors:** Weidan Xiao, Zhengmao Liu, Qijuan Zhang

**Affiliations:** ^1^ Metabolic Bone Disease Clinic The Affiliated Hospital of Wuhan Sports University Wuhan Hubei Province China; ^2^ Department of Rehabilitation Medicine The Affiliated Hospital of Wuhan Sports University Wuhan Hubei Province China

**Keywords:** apoptosis, CHON‐001 cells, inflammation, osteoarthritis, PRKACB

## Abstract

**Background:**

Previous work revealed that protein kinase cAMP‐dependent catalytic β (PRKACB) may play a crucial role in osteoarthritis (OA) development. However, the mechanism by which PRKACB plays a role in OA still needs to be further investigated. Our aim was to explore the mechanism of PRKACB in a human chondrocyte inflammatory injury model.

**Methods:**

Human CHON‐001 chondrocytes were treated with 10 ng/mL IL‐1β for 12 h to establish an in vitro model of chondrocyte inflammatory injury. Cell viability was determined by 3‐(4,5‐dimethylthiazol‐2‐yl)‐2,5‐diphenyltetrazolium bromide (MTT) assay. Flow cytometry (FCM) was conducted to assess apoptosis. Western blot assays were carried out to measure cleaved caspase‐3, caspase‐3, PRKACB, collagen II, aggrecan, phosphorated protein kinase A (p‐PKA), PKA, cAMP response element‐binding protein (CREB) and p‐CREB protein expression levels. Reverse transcription quantitative polymerase chain reaction (RT‐qPCR) assay was used to measure PRKACB gene expression. Interleukin 6 (IL‐6), IL‐1β, and tumor necrosis factor alpha (TNF‐α) levels were measured by enzyme‐linked immunosorbent assay (ELISA).

**Results:**

PRKACB expression was decreased in IL‐1β‐treated CHON‐001 cells. Transfection of a PRKACB plasmid increased PRKACB expression in CHON‐001 cells. IL‐1β significantly inhibited CHON‐001 cell viability; induced apoptosis; increased cleaved caspase‐3 expression and the cleaved caspase‐3/caspase‐3 ratio; promoted TNF‐α, IL‐6 and IL‐8 secretion; and decreased the expression levels of collagen II and aggrecan. However, these effects could be suppressed by the PRKACB plasmid. Moreover, we also found that PRKACB activated the PKA/CREB signaling pathway. H89 (a PKA inhibitor) distinctly reversed the effect of PRKACB on IL‐1β‐induced CHON‐001 cells.

**Conclusion:**

PRKACB can increase cell viability and reduce inflammation by activating the PKA/CREB signaling pathway, and PRKACB is a novel target for OA treatment.

## 1 Introduction

Osteoarthritis (OA) is the most common chronic musculoskeletal disease that occurs mainly in the elderly population, and its incidence increases with age and global aging [1, 2]. OA is the main musculoskeletal cause of mobility problems among elderly individuals and often occurs in the joints of the knees, hands, hips, and spine [3, 4]. Recent studies have revealed that, based on statistics, approximately 16% of the world's population suffers from knee OA, with a predominance of women in the prevalent population [5]. OA is clinically characterized by pain, joint stiffness, and swelling [6], and in severe stages, OA can lead to osteophytes and synovitis. In addition, over time, OA results in degeneration of the articular cartilage, loss of joint mobility and function, and impaired mobility [7]. Inflammation is now appreciated as a key pathophysiological process in OA [8, 9]. There are a number of clinical treatments currently available, including classical (nonsteroidal anti‐inflammatory drugs‐NSAIDs) and complementary (acupuncture and exercise) therapies [10, 11], biological and regenerative therapies, as well as emerging molecular approaches. Intra‐articular infiltrations (e.g., hyaluronic acid and corticosteroids) are still widely used [12]. Innovative therapies such as the combined use of stem cell therapy and platelet‐rich plasma are gradually becoming potential alternatives to traditional treatments [13]. In addition, gene therapy and molecular targeting are hotspots for scholars exploring new approaches for OA [14, 15]. For example, a recent study indicated that targeting H19 represents a new promising approach for OA treatment [16]. Currently, continuous research is needed to develop new approaches for OA.

The protein kinase cAMP‐dependent catalytic β (PRKACB) is a cAMP‐dependent protein kinase A (PKA) catalytic subunit. The PKA holoenzyme molecule is a tetramer consisting of four subunits, two of which are regulatory subunits, whereas the other two are catalytic subunits. The PRKACB gene is located on chromosome 1p31.1. PRKACB has been reported to participate in various cell development processes, including cell proliferation, differentiation, and apoptosis [17]. Furthermore, PRKACB is associated with cell growth, metabolism, and gene expression. In addition, numerous studies have shown that PRKACB serves as a key regulatory factor in multiple tumors, such as pancreatobiliary oncocytic neoplasms [18], acute myeloid leukemia [19], and colorectal carcinoma [20]. Ham demonstrated that PRKACB is a crucial factor in the PAK signaling pathway and that inhibition of the PAK pathway can effectively ameliorate OA [21]. Moreover, recent bioinformatics analysis results suggest that PRKACB plays an essential role in osteoarthritis development [22, 23]. However, the specific role of PRKACB in OA remains to be investigated. The aim of this study was to elucidate the role of PRKACB in the progression of OA by exploring the effect of PRKACB in a human chondrocyte inflammatory injury model.

## 2 Materials and Methods

### 2.1 Cell Model Construction

The chondrocyte cell line CHON‐001 was obtained from the American Type Culture Collection (ATCC). CHON‐001 cells were grown in DMEM (HyClone, Thermo Fisher) supplemented with 10% FBS (Gibco). The cells were placed in an incubator at 37°C with appropriate humidity and 5% CO_2_. To establish a chondrocyte inflammatory injury model, CHON‐001 cells were treated with 10 ng/mL IL‐1β for 12 h. We conducted subsequent experiments in IL‐1β‐induced CHON‐001 cells.

### 2.2 Cell Transfection

CHON‐001 cells were transfected with a control plasmid or a PRKACB‐expressing plasmid using Lipofectamine™ 2000 (Invitrogen). Twenty‐four hours later, the transfection efficiency was detected by RT‐qPCR and western blot analysis.

### 2.3 Reverse Transcription Quantitative Polymerase Chain Reaction (RT‐qPCR)

RNA was extracted with TRIpure Total RNA Extraction Reagent (EP013, ELK Biotechnology), and 2 μg of RNA was used as a template for reverse transcription into complementary DNA (40 ng) with EntiLink™ 1st Strand cDNA Synthesis Super Mix (EQ. 031, ELK Biotechnology). The primers were purchased from Vazyme Biotech Co. Ltd. (Vazyme). The protocol for RT–qPCR was as follows: 95°C for 5 s and 60°C for 30 s for 40 cycles. The reaction was performed with a QuantStudio 6 Flex System (Life Technologies) using EnTurbo™ SYBR Green PCR SuperMix (EQ. 001, ELK Biotechnology). GAPDH was used as a negative control. All the results were calculated using the 2^−ΔΔCt^ method.

### 2.4 Western Blot Analysis

We lysed differently treated cells with RIPA lysis buffer (AS1004, ASPEN) and collected the proteins. Proteins were separated by sodium dodecyl sulfate‐polyacrylamide gel electrophoresis (SDS‐PAGE) (10%) at a voltage of 120 V for 90 min. We then transferred the proteins to polyvinylidene difluoride (PVDF) membranes, which were then blocked with 5% skim milk powder. The membranes were washed three times with TBST. The membranes were subsequently incubated overnight at 4°C with primary antibodies against the loading control β‐actin (TDY051, 1:10,000, Beijing TDY Biotech Co. Ltd.). The primary antibodies used were as follows: anti‐PRKACB (A5324, 1:1000, ABclonal), anti‐cleaved caspase‐3 (#9661, 1:500, CST), anti‐caspase‐3 (ab32351, 1:1000, Abcam), anti‐collagen II (ab307674, 1:500, Abcam), and anti‐aggrecan (ab3778, 1: 500; Abcam). On the second day, TBST was used to wash the membranes three times. Then, we incubated the membranes with an HRP‐conjugated goat anti‐rabbit secondary antibody (AS1107/AS1106, 1:10,000, ASPEN). Finally, an ECL kit (AS1059, ASPEN) was used to detect the bands, and the data were analyzed using ImageJ software. β‐actin was used as the internal control.

### 2.5 MTT Assay

The treated cells were seeded into 96‐well plates and incubated at 37°C in a 5% CO_2_ incubator for 24 h. Then, 20 μL of MTT solution was added to each well and incubated for an additional 4 h. After the culture medium and MTT solution were removed, 150 μL of DMSO solution was added to each well to solubilize the formazan product. Finally, the absorbance value was measured at 570 nm using a microplate reader.

### 2.6 Flow Cytometry Analysis

The treated CHON‐001 cells were collected through centrifugation at 4°C. Then, a FITC/PI apoptosis detection kit (Beyotime) was used to analyze apoptosis following the manufacturer's instructions. Briefly, cells were washed, collected, and then incubated with 5 μL Annexin V‐FITC and 5 μL PI in the dark for 20 min. Finally, apoptosis was detected with a BD flow cytometer, and the data were analyzed with FlowJo software.

### 2.7 ELISA

After treatment, the cell supernatant of CHON‐001 cells was collected through centrifugation for 5 min at 400 × *g*. The levels of proinflammatory factors, including IL‐6, IL‐8, and TNF‐α, in the cell culture supernatant were determined by ELISA according to the manufacturer's instructions provided for TNF‐α (cat. no. ab181421, Abcam), IL‐8 (ab214030; Abcam), and IL‐6 (ab178013; Abcam) ELISA kits.

### 2.8 Statistical Analysis

All experiments were biologically repeated three times. The results were analyzed with GraphPad Prism software. All the data are displayed as the means ± SDs. Analysis of variance was used for comparisons among groups. *p < 0.05 and **p < 0.01 indicate statistically significant differences.

## 3 Results

### 3.1 PRKACB Expression in IL‐1β‐Induced Human Chondrocyte Models

**FIGURE 1:**
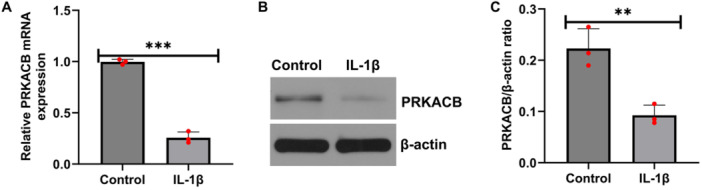
PRKACB expression was decreased in CHON‐001 cells induced by IL‐1β. (A) RT‐qPCR assay of PRKACB mRNA expression levels. (B) Western blot analysis of PRKACB protein expression levels in representative protein band images. (C) Quantitative analysis of the PRKACB protein band. *N* = 3. **p < 0.01; ***p < 0.001.

To explore the effect of PRKACB on OA, we first induced IL‐1β‐induced chondrocyte inflammatory injury in a cellular model in vitro. The model was established with CHON‐001 cells. CHON‐001 cells were treated with 10 ng/mL IL‐1β for 12 h, and RT‐qPCR and western blot assays revealed that, relative to the corresponding negative control, PRKACB expression was decreased in IL‐1β‐induced CHON‐001 cells (Figure 1A–C). These results suggest that PRKACB is downregulated in OA.

### 3.2 Transfection Efficiency of the PRKACB Plasmid in CHON‐001 Cells

**FIGURE 2:**
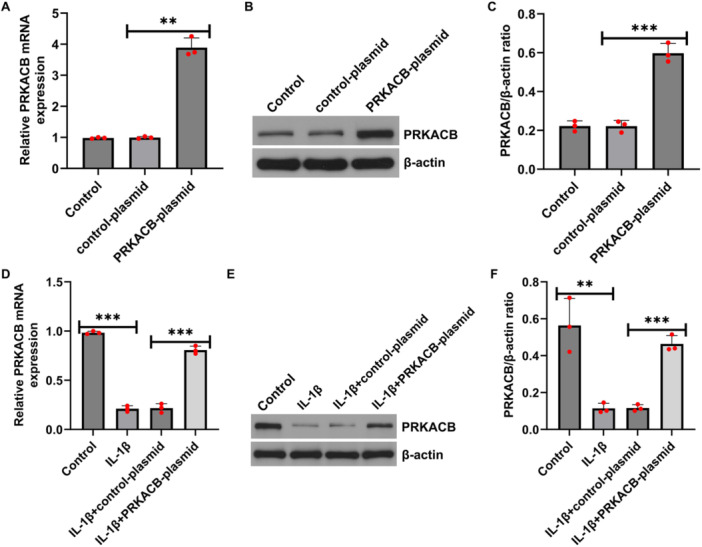
PRKACB expression was upregulated in CHON‐001 cells transfected with the PRKACB plasmid. (A) RT‐qPCR was used to detect PRKACB expression in CHON‐001 cells in the control group, control plasmid group, and PRKACB plasmid group. (B, C). Western blot analysis of PRKACB expression in representative images and quantitative analysis. RT‐qPCR (D) and western blot (E, F) analyses of PRKACB expression in CHON‐001 control, IL‐1β, IL‐1β+control plasmid, and IL‐1β+PRKACB plasmid cells. *N* = 3. **p < 0.01; ***p < 0.001.

Because PRKACB is expressed at low levels in IL‐1β‐induced CHON‐001 cells, to determine the role of PRKACB in OA, CHON‐001 cells were transfected with control plasmid or PRKACB plasmid for 24 h, and RT‐qPCR and western blot assays indicated that, relative to the control plasmid, the PRKACB plasmid notably increased PRKACB expression in CHON‐001 cells (Figure 2A–C). Then, 10 ng/mL IL‐1β was added to the transfected cells for 12 h. We further measured PRKACB expression at the mRNA and protein levels by RT‐qPCR and western blot. IL‐1β significantly reduced PRKACB expression in CHON‐001 cells compared with that in control cells, and this reduction was reversed by transfection with the PRKACB plasmid (Figure 2D–F). These data suggested that PRKACB expression was increased in the PRKACB plasmid group.

### 3.3 The Effects of PRKACB Upregulation on IL‐1β‐Induced CHON‐001 Cell Viability, Apoptosis, Inflammation and Extracellular Matrix (ECM) Degradation

**FIGURE 3:**
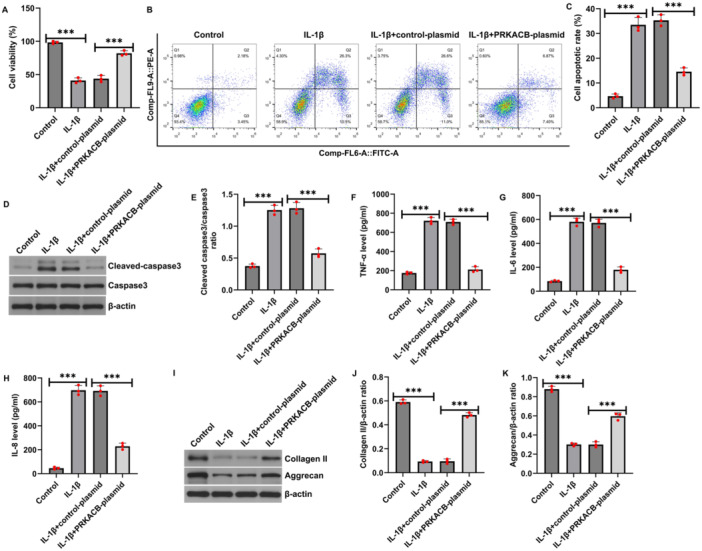
PRKACB inhibited IL‐1β‐induced CHON‐001 cell viability, apoptosis, inflammation, and ECM degradation. (A) MTT assay of CHON‐001 cell viability in the control, IL‐1β, IL‐1β+control plasmid, and IL‐1β+PRKACB plasmid groups. (B) Flow cytometry was performed to assess the degrees of apoptosis in the above four groups. (C) Quantitative analysis of apoptosis. (D) Western blot analysis of cleaved caspase‐3 expression in the above four groups. (E) Quantitative analysis of the cleaved caspase‐3/caspase‐3 ratio. (F) ELISA was carried out to detect TNF‐α expression in CHON‐001 cells in the above four groups. (G) ELISA of IL‐6 expression in CHON‐001 cells. (H) ELISA of IL‐8 expression in CHON‐001 cells. (I) Western blot analysis of collagen II and aggrecan expression. (J) Quantitative analysis of collagen II expression. (K) Quantitative analysis of aggrecan expression. *N* = 3. ***p < 0.001.

To investigate the effect of PRKACB on chondrocyte biological function, we conducted related experiments based on the transfection efficiency determined above. MTT analysis and flow cytometry revealed that, relative to the control, IL‐1β significantly inhibited CHON‐001 cell viability (Figure 3A) and induced apoptosis (Figure 3B,C). Western blot analysis also revealed that IL‐1β increased cleaved caspase‐3 expression and the cleaved caspase‐3/caspase‐3 ratio in CHON‐001 cells (Figure 3D,E). In addition, ELISA revealed that IL‐1β promoted the secretion of TNF‐α, IL‐6, and IL‐8 by CHON‐001 cells (Figure 3F–H). Western blot assays revealed that IL‐1β decreased collagen II and aggrecan expression levels (Figure 3I–K). These effects were suppressed by transfection with the PRKACB plasmid. These results indicated that PRKACB upregulation improved CHON‐001 cell viability, inhibited apoptosis, and decreased inflammation and ECM degradation.

### 3.4 Effects of PRKACB on the PKA/CREB Signaling Pathway in IL‐1β‐Induced CHON‐001 Cells

**FIGURE 4:**
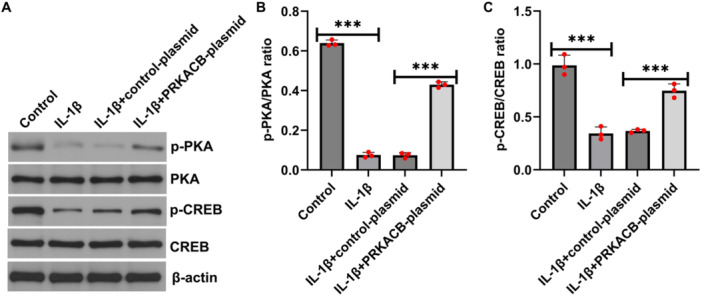
PRKACB activated the PKA/CREB signaling pathway in IL‐1β‐induced CHON‐001 cells. (A) Western blot analysis of p‐PKA, p‐CREB, PKA, and CREB expression levels. (B) Quantitative analysis of the p‐PKA/PKA ratio. (C) Quantitative analysis of the p‐CREB/CREB ratio. *N* = 3. ***p < 0.001.

To investigate the effects of PRKACB on downstream molecular regulatory mechanisms in IL‐1β‐induced human chondrocyte models in vitro, CHON‐001 cells were transfected with control plasmid or PRKACB plasmid for 24 h, and the transfected cells were treated with 10 ng/mL IL‐1β for 12 h. Western blot analysis was performed to examine the expression levels of PKA, CREB, and related proteins. Compared with the control, IL‐1β inhibited the expression of p‐PKA and p‐CREB and reduced the p‐PKA/PKA and p‐CREB/CREB ratios. These inhibitory effects were reversed by transfection with the PRKACB plasmid (Figure 4A–C). Taken together, these findings suggest that PRKACB can activate the PKA/CREB signaling pathway in IL‐1β‐induced CHON‐001 cells.

### 3.5 The Effect of H89 on the PRKACB‐Induced Activation of the PKA/CREB Signaling Pathway

**FIGURE 5:**
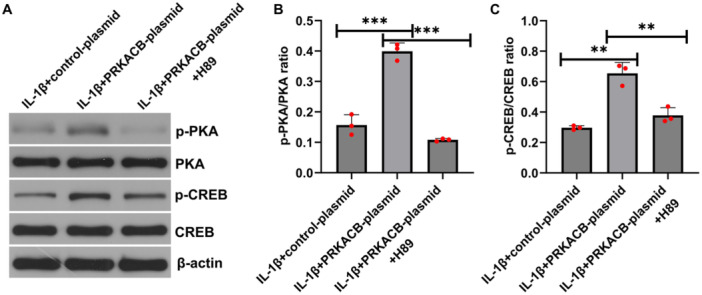
H89 suppressed the expression of p‐PKA and p‐CREB in the PRKACB‐activated PKA/CREB signaling pathway. (A) Western blot analysis of p‐PKA, p‐CREB, PKA, and CREB expression levels in the IL‐1β+control plasmid, IL‐1β+PRKACB plasmid, and IL‐1β+PRKACB plasmid+H89 groups. (B, C) Quantitative analyses of the p‐PKA/PKA and p‐CREB/CREB ratios. *N* = 3. **p < 0.01; ***p < 0.001.

H89 is an inhibitor of PKA. To explore the effect of H89 on the PRKACB‐induced activation of the PKA/CREB signaling pathway in IL‐1β‐induced CHON‐001 cells, CHON‐001 cells were transfected with the PRKACB plasmid for 24 h, and then the transfected cells were treated with 1 μM H89 for 30 min. The cells were also treated with 10 ng/mL IL‐1β for 12 h. Western blot analysis revealed that, compared with the IL‐1β+control plasmid, transfection with the PRKACB plasmid distinctly increased p‐PKA expression, p‐CREB expression, and the p‐PKA/PKA and p‐CREB/CREB ratios in CHON‐001 cells (Figure 5A–C). These improvements were inhibited by H89. Taken together, H89 blocked the PRKACB‐induced activation of the PKA/CREB signaling pathway in IL‐1β‐induced CHON‐001 cells.

### 3.6 The Effect of H89 on PRKACB Stimulates IL‐1β‐Induced CHON‐001 Cell Viability, Apoptosis, Inflammation and ECM Degradation

**FIGURE 6:**
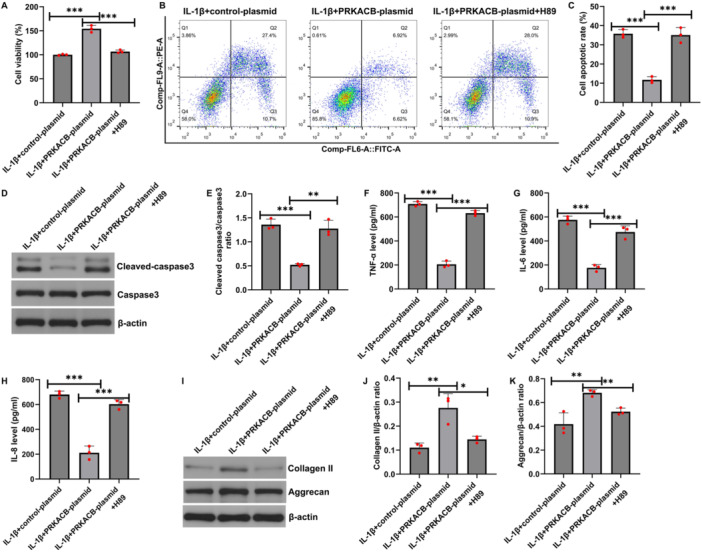
H89 suppressed cell viability, promoted apoptosis, and promoted the inflammatory response and ECM degradation. (A) MTT assay of CHON‐001 viability in the IL‐1β+control plasmid, IL‐1β+PRKACB plasmid, and IL‐1β+PRKACB plasmid+H89 groups. (B) Flow cytometry analysis of apoptosis in the above three groups. (C) Quantitative analysis of apoptosis. (D) Western blot analysis of cleaved caspase‐3 expression. (E) Quantitative analysis of the cleaved caspase‐3/caspase‐3 ratio. ELISA was carried out to detect TNF‐α (F), IL‐6 (G), and IL‐8 expression (H) in CHON‐001 cells. (I) Western blot analysis of collagen II and aggrecan expression levels. Quantitative analysis of collagen II (J) and aggrecan expression (K). *N* = 3. *p < 0.05; **p < 0.01; ***p < 0.001.

To determine the effect of H89 on the ability of PRKACB to stimulate the biological function of OA chondrocytes, we next detected cell proliferation, apoptosis, inflammatory factor secretion, and ECM‐related protein expression in IL‐1β‐induced CHON‐001 cells. Compared with the IL‐1β+control plasmid, transfection with the PRKACB plasmid increased CHON‐001 cell proliferation (Figure 6A). In contrast, FCM analysis revealed that transfection with the PRKACB plasmid decreased apoptosis (Figure 6B,C). Moreover, transfection with the PRKACB plasmid decreased cleaved caspase‐3 expression and the cleaved caspase‐3/caspase‐3 ratio, as shown by western blot analysis (Figure 6D,E). In addition, TNF‐α, IL‐6 and IL‐8 expression levels were inhibited by transfection with the PRKACB plasmid, as determined by ELISA (Figure 6F–H). Finally, collagen II and aggrecan expression levels were increased by transfection with the PRKACB plasmid, as shown by western blotting (Figure 6I–K). However, these effects were inhibited by H89. Taken together, H89 suppressed the effects of PRKACB on stimulating IL‐1β‐induced CHON‐001 cell viability, apoptosis, inflammation, and ECM degradation.

## 4 Discussion

PRKACB is a catalytic unit of PKA. A large body of evidence indicates that PRKACB is related to a variety of cancers. A previous study revealed that PRKACB participates in OA development [21]. However, the mechanism of PRKACB in OA remains unclear.

Recently, OA has attracted the attention of more scientists. OA is a global safety and health issue, with the most notable symptom being degeneration of the articular cartilage. Chondrocytes are the only cells found in articular cartilage, and some studies have shown that chondrocytes can maintain tissue homeostasis [24]. In addition, reports have indicated that joint damage in patients with OA affects chondrocyte proliferation [25, 26]. The human chondrocyte cell line CHON‐001 is obtained from normal human articular cartilage, is currently selected for many OA studies, and is used with IL‐1β induction as an in vitro OA cell model [27, 28, 29]. Li et al. reported that inflammation can induce OA occurrence and development, the severity of OA is positively correlated with the expression of the proinflammatory factor IL‐1β, and microRNA‐186‐5p downregulation suppresses the OA development process [30]. Combining the findings from multiple studies in the literature, we established a cellular model of OA in vitro and treated CHON‐001 cells with IL‐1β. In our study, we investigated the effects of PRKACB on a model of inflammatory injury in human chondrocytes. Our results revealed that PRKACB expression was decreased in IL‐1β‐induced CHON‐001 cells.

Guo et al. reported that different concentrations of IL‐1β inhibited CHON‐001 cells in a concentration‐dependent manner and that IL‐1β promoted apoptosis [31]. Moreover, Zuo's team reported that IL‐1β could suppress cell proliferation and induce apoptosis [32]. Our findings are consistent with these reports. Our results indicated that IL‐1β inhibited CHON‐001 cell viability and induced apoptosis. In addition, we explored the effects of IL‐1β on apoptosis‐related proteins. We found that IL‐1β could increase cleaved caspase‐3 expression. Weber et al. reported that the proinflammatory factors TNF‐α, IL‐6, and IL‐8 are associated with OA [33]. Many studies have indicated that IL‐1β promotes the expression of the proinflammatory factors TNF‐α, IL‐6, and IL‐8 [31, 32, 33]. Our experimental results led to the same conclusions with respect to the expression of proinflammatory factors.

Articular cartilage consists of a small number of unique chondrocytes and a large amount of ECM. The role of the ECM is to maintain the cartilage structure and balance the extracellular environment of chondrocytes [34]. The components of the ECM are mainly type II collagen and aggrecan. When patients suffer from OA, cartilage function is abnormal, mainly due to the degradation and inhibition of ECM synthesis [35]. Several studies have shown that IL‐1β can cause ECM degradation by promoting the synthesis of ECM‐degrading enzymes such as collagenase and aggrecanase [36, 37]. In addition, IL‐1β also reduces ECM synthesis by suppressing the expression of related genes [38, 39]. Similarly, Zhuang et al. revealed that IL‐1β inhibited collagen II and aggrecan expression [40]. Our results demonstrated that IL‐1β decreased collagen II and aggrecan expression. Moreover, our results revealed that transfection with the PRKACB plasmid inhibited the effect of IL‐1β on CHON‐001 cells, as PRKACB upregulation promoted cell viability, inhibited apoptosis, decreased cleaved caspase‐3 expression, suppressed proinflammatory factor expression, and increased collagen II and aggrecan expression in CHON‐001 cells.

Wang et al. reported that OA development is related to the protein kinase A/cAMP‐response element‐binding protein (PKA/CREB) signaling pathway [41]. Our results indicated that IL‐1β inhibited p‐PKA and p‐CREB expression and that transfection with the PRKACB plasmid reversed the inhibitory effect of IL‐1β. Transfection with the PRKACB plasmid also improved p‐PKA and p‐CREB expression. H89, a small‐molecule protein kinase A (PKA) inhibitor, is related to the chondrocyte and PKA signaling pathways [21]. Protein kinase A is involved in a variety of cellular processes. PKA function has been studied mainly using pharmacological inhibitors. H89 is a small molecule that can easily cross cell membranes to exert its inhibitory effects [42, 43]. Our study demonstrated that H89 blocked the PKA/CREB signaling pathway to inhibit CHON‐001 cell viability, promote apoptosis, increase proinflammatory factor expression, and decrease ECM degradation.

There were also some limitations of this study. First, this study is an in vitro cell experiment and lacks validation in animal models. Secondly, whether other downstream pathways or genes are involved in the role of PRKACB in OA cell models still requires further investigation. Finally, this study lacks clinical validation. We will perform these issues in the future.

## 5 Conclusion

PRKACB is a potential therapeutic target for osteoarthritis, as it attenuates chondrocyte loss and the inflammatory response through activating the PKA/CREB pathway in bone joints.

## Author Contributions

Weidan Xiao contributed to the study design, data collection, statistical analysis, data interpretation, and manuscript preparation. Zhengmao Liu contributed to data collection and statistical analysis. Qijuan Zhang contributed to data collection and manuscript preparation. All authors read and approved the final manuscript.

## Ethics Statement

The authors have nothing to report.

## Consent

The authors have nothing to report.

## Conflicts of Interest

The authors declare no conflicts of interest.

## Data Availability

The datasets used and/or analyzed during the current study are available from the corresponding author on reasonable request.
